# P02.110. Recruitment experiences from piloting the LEAP project: an online spirituality based depression intervention for young adults

**DOI:** 10.1186/1472-6882-12-S1-P166

**Published:** 2012-06-12

**Authors:** P Paccagnan, S Moritz, N Rickhi, C Dennis, S Malhotra, C Hart, R Maser, B Rickhi, J Toews, J Cohen

**Affiliations:** 1 The Canadian Institute of Natural and Integrative Medicine, Calgary, Canada; 2 Department of Psychiatry, University of Calgary, Calgary, Canada

## Purpose

It is a challenge to recruit young individuals with mental health problems to a clinical trial. Here we describe the recruitment efforts and resulting enrollment for recruiting depressed adolescents to a community based clinical trial that piloted an 8-week, online, spirituality-based intervention (http://www.leapproject.com).

## Methods

This trial aimed to recruit adolescents suffering from mild to moderate unipolar depression. A multi-faceted recruitment strategy was used. Poor enrolment during the first 13 months (Phase I) prompted a review of eligibility criteria. Revised eligibility criteria included an age expansion from previously 13-18 to 13-24 and allowance of general, non-depression specific, counselling. An observational study was conducted to assess the impact of investing trial resources into the various recruitment sources pre (Phase I = 13 month) and post (Phase II = 10 months) eligibility change.

## Results

In total, 1667 staff hours (mail outs, emails, presentations and personal contacts) were invested to generate 196 referrals from five different referral sources (Health Care Professionals, High Schools, Post Secondary institutions and Public Advertising). In Phase I, 11 participants were enrolled (13% enrollment rate) compared to 27 participants in Phase II (25% enrollment rate). In Phase I, invested staff hours to generate a referral that would result in a successful enrollment (82 hours/participant) were three fold that required in Phase II (28 hours/participant). Health Care Professionals and Post Secondary Institutions generated the largest number of referrals and had the highest enrolment rates.

## Conclusion

Our findings suggest that depressed young people can be successfully recruited to a clinical trial. Eligibility changes accommodated the available client base better, resulting in more referrals and fewer failed eligibility assessments. Recruitment was boosted significantly through referrals from post secondary institutions and through building relationships with health care professionals.

